# Noninvasive prediction of axillary lymph node breast cancer metastasis using morphometric analysis of nodal tumor microvessels in a contrast-free ultrasound approach

**DOI:** 10.1186/s13058-023-01670-z

**Published:** 2023-06-09

**Authors:** Giulia Ferroni, Soroosh Sabeti, Tasneem Abdus-Shakur, Lorenzo Scalise, Jodi M. Carter, Robert T. Fazzio, Nicholas B. Larson, Mostafa Fatemi, Azra Alizad

**Affiliations:** 1 Department of Physiology and Biomedical Engineering, Mayo Clinic College of Medicine and Science, Rochester, MN 55905 USA; 2 Department of Radiology, Mayo Clinic College of Medicine and Science, 200 1st. St. SW, Rochester, MN 55905 USA; 3 Department of Industrial Engineering and Mathematical Science, Marche Polytechnic University, 60131 Ancona, Italy; 4 Department of Laboratory Medicine and Pathology, Mayo Clinic College of Medicine and Science, Rochester, MN USA; 5 Department of Laboratory Medicine and Pathology, University of Alberta, Edmonton, AB Canada; 6 Department of Quantitative Health Sciences, Mayo Clinic College of Medicine and Science, Rochester, MN 55905 USA

**Keywords:** Breast cancer, Ultrasound microvessel imaging, Axillary lymph node metastasis, Flow imaging, Vessel morphological biomarkers

## Abstract

**Purpose:**

Changes in microcirculation of axillary lymph nodes (ALNs) may indicate metastasis. Reliable noninvasive imaging technique to quantify such variations is lacking. We aim to develop and investigate a contrast-free ultrasound quantitative microvasculature imaging technique for detection of metastatic ALN in vivo*.*

**Experimental design:**

The proposed ultrasound-based technique, high-definition microvasculature imaging (HDMI) provides superb images of tumor microvasculature at sub-millimeter size scales and enables quantitative analysis of microvessels structures. We evaluated the new HDMI technique on 68 breast cancer patients with ultrasound-identified suspicious ipsilateral axillary lymph nodes recommended for fine needle aspiration biopsy (FNAB). HDMI was conducted before the FNAB and vessel morphological features were extracted, analyzed, and the results were correlated with the histopathology*.*

**Results:**

Out of 15 evaluated quantitative HDMI biomarkers, 11 were significantly different in metastatic and reactive ALNs (10 with *P* << 0.01 and one with 0.01 < *P* < 0.05). We further showed that through analysis of these biomarkers, a predictive model trained on HDMI biomarkers combined with clinical information (i.e., age, node size, cortical thickness, and BI-RADS score) could identify metastatic lymph nodes with an area under the curve of 0.9 (95% CI [0.82,0.98]), sensitivity of 90%, and specificity of 88%.

**Conclusions:**

The promising results of our morphometric analysis of HDMI on ALNs offer a new means of detecting lymph node metastasis when used as a complementary imaging tool to conventional ultrasound. The fact that it does not require injection of contrast agents simplifies its use in routine clinical practice.

## Introduction

Axillary lymph nodes (ALNs) status is a key predictive factor for distant metastasis and recurrence risk in breast cancer patients [1]. A correct assessment of the status of ALNs is crucial for staging breast cancer and selection of appropriate treatment option. Physical examination, such as palpation of the axilla, is notoriously inaccurate and axillary metastasis estimated by clinical palpation is associated with a large proportion of false-negative rates (%70) and a false-positive rate of 20% [2]. Visualization of ALN is limited in digital mammography or digital breast tomosynthesis, particularly for deeper seated level I and level II nodes [3]. Among the current imaging modalities, ultrasound (US) is the primary method to evaluate ALNs in women with newly diagnosed breast cancer, and the number of ALN metastases determined by preoperative ultrasound can be useful in predicting prognosis [4]. However, wide ranges of sensitivity (49–87%) and specificity (55–97%) have been reported [5, 6]. The addition of shear wave elastography to conventional US has relatively increased the sensitivity and specificity of ultrasound for breast cancer detection [7, 8], as well as improved prediction of cancer invasiveness and identification of metastatic ALNs [9–11]. Traditionally, preoperative identification of axillary metastases is through US-guided biopsy as well as sentinel lymph node biopsy and surgical excision, which is associated with complications such as infection as well as long term problems including lymphedema, localized swelling, sensory loss, and weakness [12–15]. Therefore, a preoperative and noninvasive alternative approach is essential to accurately identify and quantify metastatic ALNs.

Angiogenesis, or the formation of new blood vessels, is a critical component of progression of the tumor growth and metastasis [16, 17] and has a crucial role in prediction of cancer aggressiveness and poor prognosis [18]. Studies have suggested that angiogenic activity in metastatic tumors, rather than primary tumors, may better predict prognosis in individuals with breast cancer [18, 19]. Tumor angiogenesis is generally measured by quantifying microvessel density (MVD). MVD is positively correlated with prognosis, histological grade, and lymph node status [20]. Moreover, morphological features of tumor microvessels are expected to be important biomarkers in differentiating cancerous from non-cancerous masses [21, 22]. Evaluating the vascularity of ALNs by noninvasive techniques could be useful in predicting lymph node metastasis, especially in the absence of typical sonographic findings.

Only few imaging techniques are capable of visualizing vascularity with tumor size scale and penetration depth. Dynamic contrast-enhanced magnetic resonance imaging (DCE-MRI) may provide noninvasive kinetic information suggesting axillary nodal status, but it has lower specificity; also, it is not affordable or widely available [23]. Conventional Doppler US is also useful for identifying nonhilar peripheral blood flow seen in metastatic ALNs [24], but it only detects rapid flow, producing highly fragmented, patchy images of vasculature, making the structural analysis of microvessels impossible. Other imaging modalities, such as Photoacoustic imaging, have been employed to visualize microvasculature structure [25]; though, it is only applicable for superficial tumors [26]. Recently, superb microvascular imaging (SMI) [27] and microflow imaging [28] modalities have been investigated for detecting metastatic ALN, without using a contrast agent; however, the diagnosis is mostly based on pixel counting and visual inspection.

Recently, a novel contrast-free ultrasound-based modality has been developed to visualize sub-millimeter vessels as small as 300 μm in diameter [29], named high-definition microvessel imaging (HDMI) [30]. The HDMI approach is equipped with a series of morphological filtering and vessel enhancement in order to quantify tumor vessel morphological parameters as quantitative vessel biomarkers [31, 32]. The performance of quantitative HDMI biomarkers has been reported for differentiation of benign and malignant breast masses [30], thyroid nodules [33], and hepatic masses [34]. We hypothesize that morphometric analysis of node microvessels can objectively distinguish metastatic axillary lymph nodes from reactive, thus rendering this method less operator-dependent and eliminating the observer/reader variability for reliable clinical use. To test this hypothesis, gross images of ALN microvasculature and vessel quantitative biomarkers are first derived using the HDMI technique and quantification algorithms [29, 31, 32], to depict the microvascular architecture of ALNs. Furthermore, all the vessel biomarkers are combined to create a model capable of classifying the ALN as metastatic or reactive.

## Materials and methods

### Ethics approval

This prospective single-center study, from June 2018 to October 2022, was approved by the institutional review board (IRB#13-006035 and IRB # 19-003028) and was in accordance with the Health Insurance Portability and Accountability Act. Prior to the study, each enrolled participant signed an IRB approved written informed consent with permission for publication.

### Study population

The imaging study was completed on 71 female volunteers with recently diagnosed breast cancer and with suspicious ALNs in clinical ultrasound imaging. The enrolled patients were previously assigned the Breast Imaging Reporting and Data System (BI-RADS) assessments for breast lesions by their radiologists and referred for breast core needle biopsy as their clinical plan; therefore, BI-RADS scores of 4, 5 or 6 were included. Inclusion criteria include patients ages 18 and up who had a suspicious axillary lymph node identified by axillary ultrasound and were scheduled for ultrasound guided fine needle aspiration biopsy (FNAB) of ALN, as part of their clinical care. A total of three patients were excluded from the data analysis. Among excluded patients, one scheduled FNAB was cancelled due to benign appearance of the lymph node at the second look in ultrasound imaging performed just before the procedure, the report of surgery indicated that 0 of 1 sentinel lymph nodes were positive for metastatic disease. Other excluded patients (*N* = 2) preferred to have FNAB and follow-ups in their hometown. The final cohort included 68 female patients, age 28–88 years (mean ± standard deviation: 55.7 ± 12.9 y). Patients did not receive any treatment before the study, and the pathology results of the FNAB, obtained from the clinical record, were used as the reference gold standard. All quantitative HDMI studies were conducted prior to FNAB.

### Clinical ultrasound features

All enrolled patients had clinical breast imaging examinations and were assigned BI-RADS scores for breast lesions before the breast cancer diagnosis. The axilla was imaged with ultrasound if a breast mass fell into BIRADS 4/5 or if a breast mass was biopsied and positive for malignancy. Subsequently, our study patients underwent axillary ultrasound examination as their clinical care and the morphological features of lymph nodes were assessed. Among the ultrasound features of ALNs, the cortical thickness and the presence or absence of a fatty hilum were considered in analysis as clinical imaging findings.

### High-definition microvasculature imaging and extraction of vessel biomarkers

The imaging studies were performed using an Alpinion Ecube12-R ultrasound machine (ALPINION Medical Systems, Seoul, Korea) with capability of plane wave imaging providing a sequence of high-frame-rate images. A linear array transducer, L3-12H operating at 8.5 MHz (ALPINION Medical Systems, Seoul, South Korea) was used for studying subjects with ALNs. Two sonographers with more than 30 and 18 years of ultrasound scanning experience, conducted the US examination. The axillary lymph nodes were identified on B-mode plane wave imaging mode and a sequence of high-frame-rate data (at ~ 600 frames per second) was acquired on the ALN site, as detailed in [30]. To reduce undesired compression effect on altering tissue microvessel, our sonographers were instructed to lower the preload during ultrasound examination. To minimize motion artifacts, patients were asked to remain still and hold their breath for around 3 s while the data was being collected. Two acquisitions were made for each orientation of the HDMI scan to improve repeatability. Only one of the two sonographers participated in the HDMI scanning for each patient participant. HDMI image processing and denoising have been done as reported in [8, 35–39]. The ALNs were manually segmented using B-mode images obtained from the IQ data reconstruction, binary and skeleton images were produced to quantify morphological parameters of tumor microvessels [32].

### Microvessel morphometric analysis

**Fig. 1:**
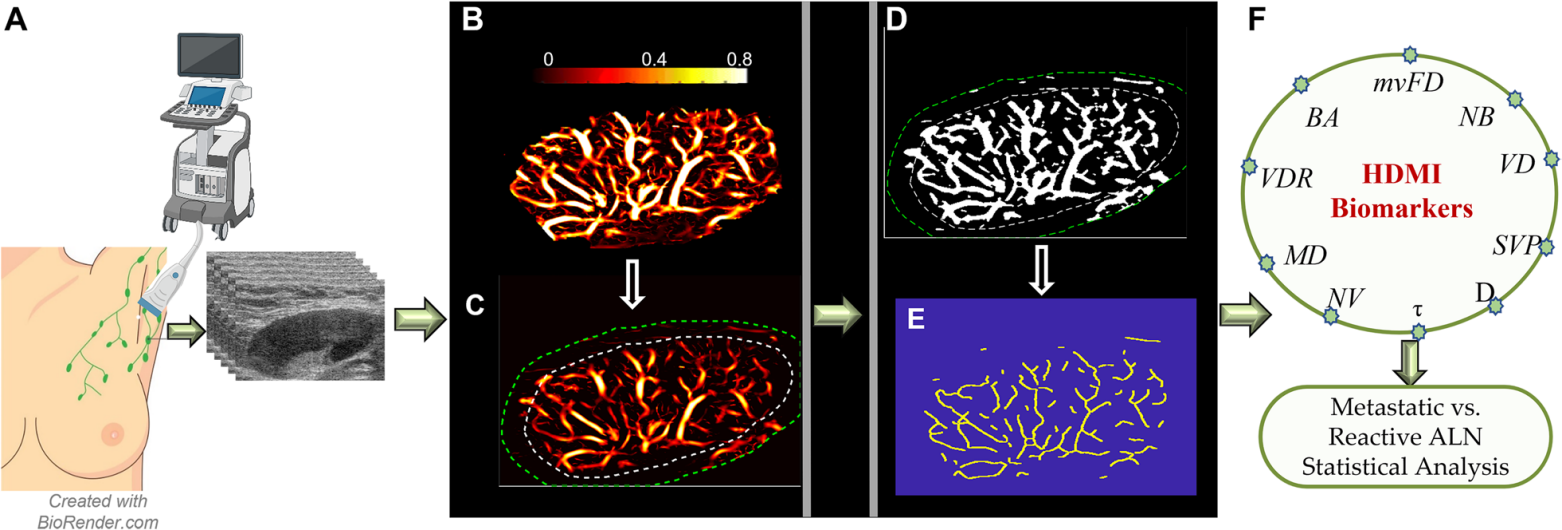
HDMI image acquisition and a set of processing for image segmentation to extract vessel biomarkers: **A** HDMI image acquisition of ALN, **B** Microvasculature image of a metastatic ALN, **C** Defined ROI with 2 mm dilation of the ALN, **D** Conversion of the microvasculature image into a binary image, **E** skeleton image of vessel network, **F** HDMI biomarkers extracted for the skeleton to be used for distinguishing the metastatic ALN from reactive. HDMI: High definition microvessel imaging; ALN: Axillary lymph node; mvFD: Microvessel fractal dimension; NB: Number of branch points; VD: Vessel density; SVP: Spatial vascularity pattern; D: Diameter; *τ:* Tortuosity; NV: Number of vessel segments; MD: Murray’s deviation; VDR: Vessel density ratio; BA: Bifurcation angle.

For each targeted lymph node, a region of interest (ROI) was defined based on the cortex and hilum boundaries acquired from the B-mode ultrasound images. To include peripheral vascularity, the defined ROI was dilated 2 mm. After the image formation process, a set of processing steps consisting of converting to binary image and constructing the full skeleton of the vessel network, were performed to prepare the microvessel images for quantification of morphological parameters [32]. After all these steps, analysis of desired quantitative parameters of the vessels has been done on the vessel segments of output images. A series of microvessel morphological parameters of lymph node including, number of vessel segments (*NV*), number of branch points (*NB*), vessel density (*VD*), vessel diameter (*D*), distance metric as a measure of tortuosity (*τ*), microvessel fractal dimension (*mvFD*), Murray’s deviation (*MD*), bifurcation angle (*BA*), and spatial vascularity pattern (*SVP*) calculated by vessel density ratio (*VDR*), were extracted from output HDMI images, and measured. The methods for obtaining HDMI images, vessel extraction and steps for vessel segmentation have been detailed in [8, 30–32]. A diagram presenting HDMI image acquisition, image formation and segmentation to extract the vessel biomarkers is shown in Fig. 1.

### Microvessel morphological parameters

The tumor microvessel morphological parameters extracted from the HDMI images were used in this study as imaging biomarkers*.* One of the most known parameters obtained through HDMI is the vessel density, defined as the ratio of the geometric area of vessel segments to the geometric area of the associated lesion region of interest [40]. Moreover, we have also calculated the number of vessel segments (*NV*), and the number of branch points (*NB*) (branch point defined as a common point connected to three or more vessel segments) [32, 41]. Vessel diameter, defined as two times the minimum distance between the vessel centerline and the vessel border has also been measured [32]. Furthermore, Murray’s deviation (*MD*) was an important biomarker used in the analysis. Murray’s deviation presents a diameter mismatch, defined as the deviations from Murray’s Law, definition, and calculation of which has been detailed in [31]. Additionally**,** vessel tortuosity*,* determined by the distance metric (*DM*), which is defined as the ratio between the actual path length of a meandering curve (vessel) and the linear distance between the two endpoints [32], was evaluated in this study.

Another biomarker that has been included in the analysis is microvessel fractal dimension* (mvFD*)*,* a unit-less, geometrical feature to quantify the structural complexity of a vascular network to provide additional diagnostic and prognostic information [31, 42]. Moreover, bifurcation angle, that refers to the angle between two daughter vessels, has also been used as a distinctive biomarker, detailed definition and calculation of which can be found in [31]. Vessel density ratio *(VDR),* defined as the ratio of vessel density of the tumor center to periphery [31], and spatial vascularity pattern (*SVP*), calculated by *VDR*, can present the tumor vascular distribution pattern as being either intratumoral or being peritumoral [31, 43].

The proposed morphological operations and quantification steps have been well detailed in our previous papers [31, 32].

### Fine needle aspiration biopsy

All study patients underwent FNAB within one hour after the HDMI test. In our institution the false-negative rate of FNAB is very low. Infrequently, core needle biopsy is conducted for any discordant or non-diagnostic FNA results. FNAB was done by one of our six board-certified radiologists with 10 to 30 years of experience in breast imaging. The procedure was done under ultrasound guidance and standard sterile technique, using a 25-gauge needle to obtain six fine needle aspirates for each ALN. Slides were immediately prepared and sent for cytology. Specimens underwent routine pathologic examination and results were obtained from the clinical record. The pathological results of FNAB for all ALNs, positive or negative were included for data analysis as reference gold standard.

### Statistical analysis and classification modeling

All images and data were analyzed by the members of our investigative team who were blinded to the results of ALN biopsy. All statistical analyses were performed using MATLAB® environment (MATLAB version R2022b). For each image, vascular morphological features were examined for statistical significance in distinguishing metastatic from reactive lymph nodes, using pathology outcomes as the reference gold standard. Box plots were created for each quantitative biomarker and the Wilcoxon rank-sum test was performed to evaluate distributional differences by malignancy status. Statistical significance was considered with *p* < 0.05.

To evaluate the collective discrimination information of the HDMI biomarkers for ALN malignancy status, we trained multivariable machine learning classification models. The training of the classifier was implemented in Classification Learner app on MATLAB. This app allows performing supervised machine learning by supplying a known set of input data and corresponding known labels. Three models were developed in this framework. First, all of the HDMI biomarkers were used to train a classification model, called HDMI model, to classify the metastatic and reactive ALNs. Next, clinical factors, namely age, cortical thickness, lymph node size, and BI-RADS were added to the HDMI model. The new model was denoted as the HDMI-C model. Finally, a model trained only on the aforementioned clinical factors was developed and was named the C model. In order to avoid the problem of overfitting, the training was performed using fivefold cross-validation. The modeling was performed using a random 70/30 train/test split (48 training samples, and 20 test (10 metastatic, and 10 reactive) samples) using a support vector machine (SVM) algorithm with a coarse Gaussian kernel, which was then applied to the leave-out test data for performance evaluation. SVM is a common and efficient supervised machine learning algorithm utilized for classification purposes. The performances of trained models on the test set were summarized using receiver operating characteristic (ROC) curve analysis, reporting the area under the ROC curve (AUC) and the corresponding 95% confidence interval (CI). From the resulting ROCs, cut-off thresholds corresponding to the points closest to maximum sensitivity and specificity (top-left corner) were selected to generate estimates of sensitivity, specificity, and accuracy.

#### Sample size and statistical power

This study was not designed to be powered to detect a specific effect size a priori. The analytical cohort sample size after consideration for inclusion/exclusion criteria was *N* = 68 (34 reactive, 34 metastatic). Under these conditions, a two-sample Wilcoxon rank-sum test for mean differences in biomarker values by malignancy status under a two-sided alpha level of 0.05 would have approximately > 90% power to identify a difference equivalent to 0.82 standard deviations, per simulation under assumptions of equal variance and normality.

## Results

Of the total of 68 patients, the pathology data of FNAB indicated that 34 ALNs were negative for metastasis and 34 were positive for metastatic carcinoma. Of the metastatic lymph nodes, the primary tumors were mostly classified as BI-RADS 5 and 6, 38%, and 47%, respectively. The most common histological type of these primary tumors was invasive ductal carcinoma (79%) and most were reported as grade 2 (56%) and grade 3 (38%). Details of the primary tumors for metastatic lymph nodes are shown in Table 1.

**Table 1:** Details of the primary tumors for metastatic lymph nodes

Metastatic *n* = 34 (50%)	
Malignant grade	
1	2 (6%)
2	19 (56%)
3	13 (38%)
ER (positive)	31 (91%)
PR (positive)	28 (82%)
HER2 (positive)	10 (29%)
Ki-67^a^	0.25 ± 0.17
BIRADS	
4	5 (15%)
5	13 (38%)
6	16 (47%)
Histological Type	
Invasive ductal carcinoma	27 (79%)
Invasive lobular carcinoma	5 (15%)
Invasive mammary carcinoma with mixed ductal and lobular features	2 (6%)

^a^Numbers are presented in mean ± standard deviation

The cortical thickness ranged from 2 to 25 mm, with a mean ± standard deviation of 6 ± 4 mm. The lymph node size ranged from 13 to 41 mm, with a mean ± standard deviation of 23 ± 6 mm. Table 2 summarizes the relevant results of patient and lymph node characteristics as well as the results of quantitative HDMI biomarkers and respective p-values for reactive and metastatic ALN. The values of the number of vessel segments (NV), number of branch points (NB), vessel density (VD), maximum tortuosity (*τ*_max_)*,* microvessel fractal dimension (mvFD), maximum Murray’s deviation (MD_max_)*,* and maximum diameter (D_max_) were much higher in the metastatic nodes compared to the reactive ones. Mean bifurcation angle (BA_mean_) was lower in the metastatic ALN.

**Table 2:** Participant demographics, lymph node characteristics, and the summary of the performance of quantitative HDMI biomarkers

	Reactive (*n* = 34)	Metastatic (*n* = 34)	*p-*value^b^
Age (y)	55.06 ± 12.93	56.6 ± 13.04	0.73
ALN size (mm)	22 ± 6	24 ± 6	**0.0135**
Cortical thickness (mm)	4.8 ± 2.0	7.7 ± 4.4	** < 0.001**
HDMI Biomarkers			
(DM_mean_)*τ*_mean_	1.03 ± 0.03	1.04 ± 0.02	**0.005**
(DM_max_)*τ*_max_	1.14 ± 0.16	1.27 ± 0.22	**0.00017**
NV	9.56 ± 7.96	24.65 ± 23.13	** < 0.00001**
NB	4.00 ± 1.19	13.59 ± 16.44	**0.00001**
VD	0.03 ± 0.02	0.06 ± 0.04	**0.00018**
VDR	4.94 ± 4.84	2.35 ± 1.73	**0.02**
mvFD	1.17 ± 0.11	1.29 ± 0.10	** < 0.00001**
μm D_max_	548.73 ± 106.89	566.03 ± 0.38	0.13
μm D_mean_	450.41 ± 86.49	449.39 ± 0.31	0.49
MD_max_	0.46 ± 0.27	0.64 ± 0.23	**0.007**
MD_mean_	0.31 ± 0.16	0.32 ± 0.07	0.40
MD_min_	0.18 ± 0.17	0.08 ± 0.09	**0.003**
BA_max_	114.63 ± 23.60	135.40 ± 11.24	**0.006**
BA_mean_	101.28 ± 18.36	101.03 ± 11.24	0.92
BA_min_	85.37 ± 27.73	62.80 ± 28.39	**0.004**

*Unless otherwise specified, Data are presented as mean* ± *standard deviation*

ª Data are numbers of participants

^b^ Wilcoxon rank-sum test

Numbers in bold indicate statistical significance, i.e., p-value < 0.05

### High-definition microvasculature imaging of representative metastatic and reactive ALN cases

**Fig. 2:**
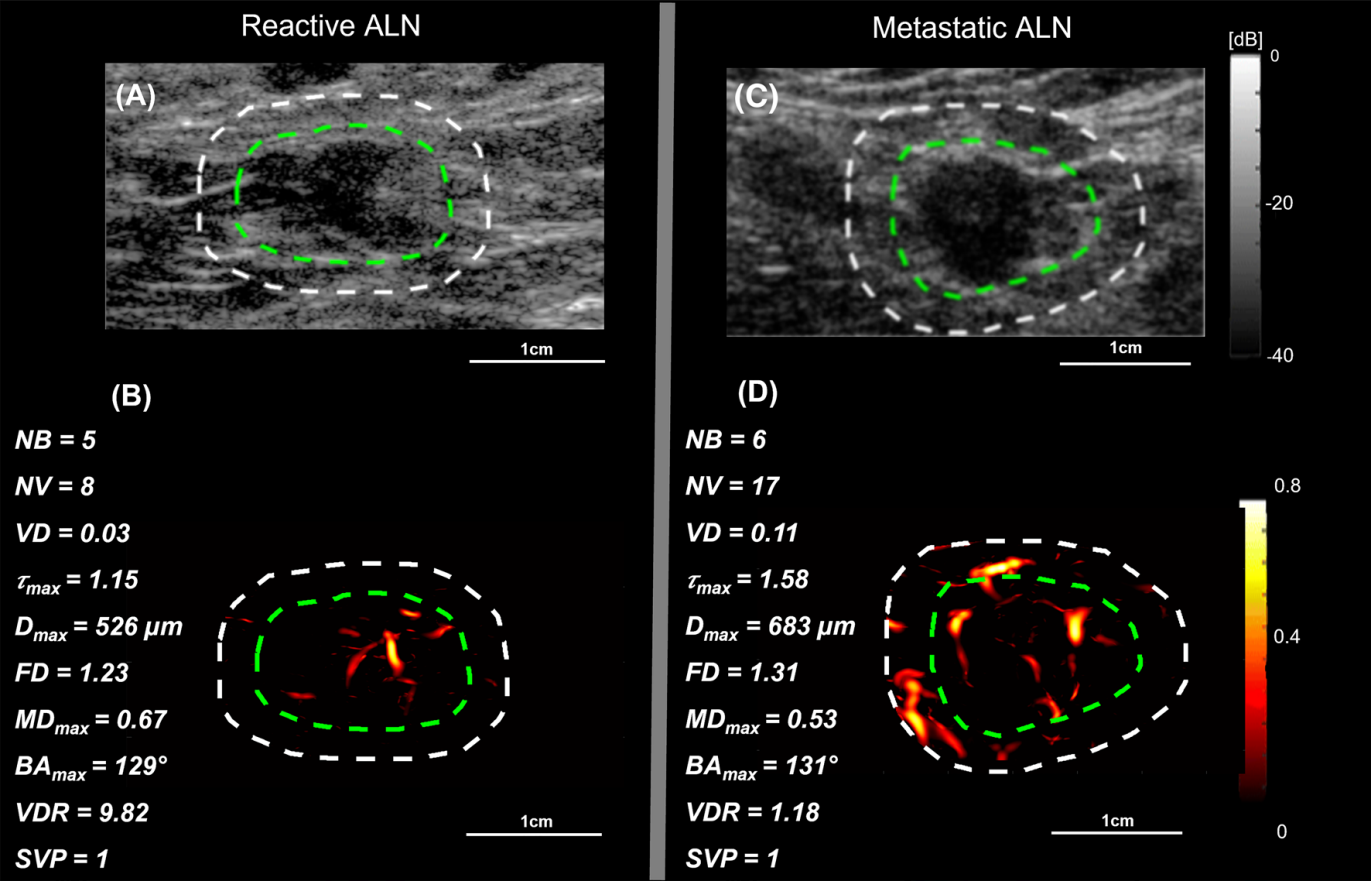
B-mode and HDMI images of small metastatic and reactive ALNs. The relative quantitative biomarkers are shown on the left side of each HDMI image. **A** and **B** are B-mode and HDMI images of a small reactive ALN in a 39-year-old woman, respectively. **C** and **D** are B-mode and HDMI images of a small metastatic ALN in a 60-year-old woman, respectively. The dashed white and green curves indicate the boundaries of ALNs with and without the 2 mm dilation, respectively

**Fig. 3:**
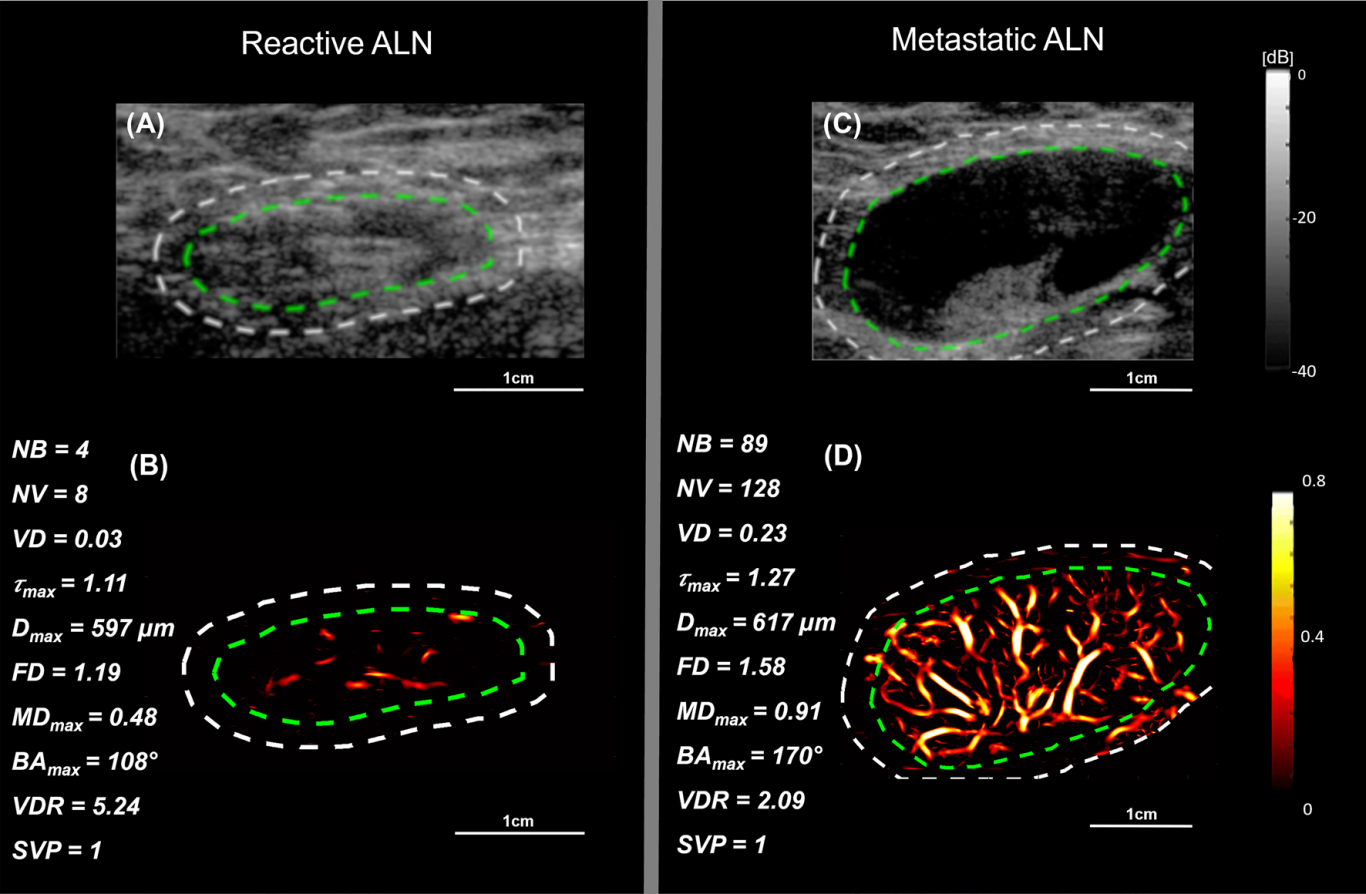
B-mode and HDMI images of large metastatic and reactive ALNs. The relative quantitative biomarkers are shown on the left side for each HDMI image. **A** and **B** are B-mode and HDMI images of a large reactive ALN in a 41-year-old woman, respectively. **C** and **D** are B-mode and HDMI images of a metastatic ALN in a 63-year-old woman, respectively. The dashed white and green curves indicate the boundaries of ALNs with and without the 2 mm dilation, respectively

We demonstrated the visual presentation of the conventional B-mode and the HDMI images of two reactive and two metastatic ALNs along with the values of respective HDMI biomarkers in Figs. 2 and 3. Figure 2 depicts the B-mode and HDMI images of small size lymph nodes, 16 mm in reactive and 14 mm in metastatic, in largest dimension. The visual inspection of the metastatic ALN displays more vascularity with dilated vessels than in reactive. The quantitative HDMI biomarkers shown on the sides of each HDMI image represent the differences in the values of each biomarker for reactive and metastatic lymph nodes. Figure 3 illustrates the B-mode and HDMI images of larger lymph nodes, with a mass size along the largest dimension of 25 mm in reactive and 30 mm in metastatic. Visual inspection shows considerable hypervascularity and morphological irregularity of microvessels in metastatic ALN compared to reactive that has fewer microvessels.

### Statistical results of HDMI biomarkers for differentiation of metastatic and reactive ALNs

**Fig. 4:**
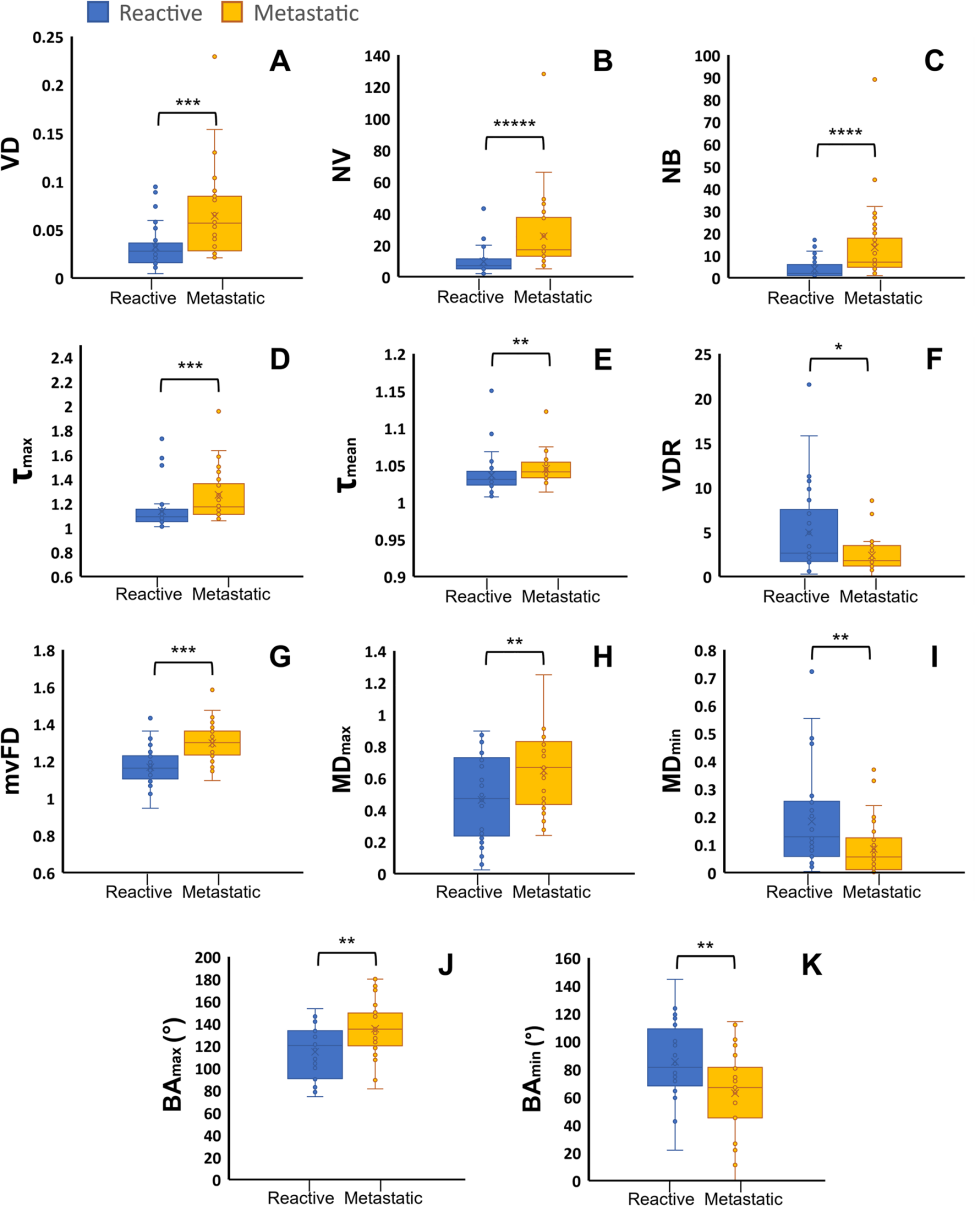
Box plots of significant biomarkers for differentiation of reactive and metastatic lymph nodes. **A**. Vessel density (VD), **B**. Number of vessel segments (NV), **C.** Number of branch points (NB), **D**. Maximum tortuosity (τ_max_), **E**. Mean tortuosity (τ_mean_), **F**. Vessel density ratio (VDR), G. Microvessel fractal dimension (mvFD**), H.** Maximum Murray’s deviation (MD_max_), **I.** Minimum Murray’s deviation (MD_min_), **J**. Maximum bifurcation angle (BA_max_), **K**. Minimum bifurcation angle (BA_min_). *p < 0.05, **p < 0.01, ***p < 0.001, **** < 0.0001, ***** < 0.00001, Benign (*n* = 34) Malignant (*n* = 34)

Most of quantitative HDMI biomarkers (11/15) show statistically significant differences at *p* < 0.05 between metastatic and reactive ALNs, as reported in Table 2. The values of *NV, NB, VD*, *mvFD*, *τ*_max_ were significantly higher for metastatic ALNs with *p*-values of < 0.001. The values of *BA*_max_ and *BA*_min_ showed significant differences between reactive and metastatic with *p*-values of 0.006 and 0.004, respectively. Figure 4A–K shows the boxplots for VD, NB, NV, *τ*_max_*,* *τ*_mean_*, *VDR, mvFD, MD_max_*, *MD_min_*, *BA_max_*,* and BA_min_*.*

**Fig. 5:**
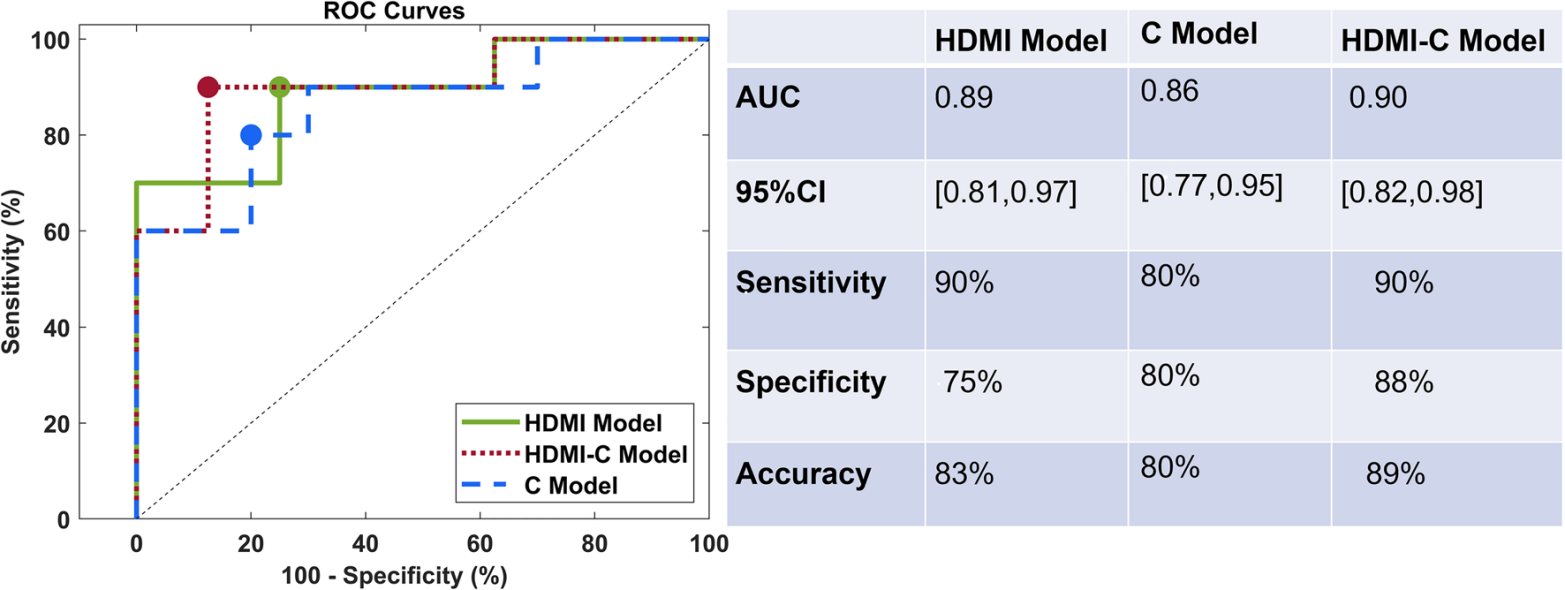
ROC curves generated with HDMI biomarkers extracted from HDMI images, and clinical biomarkers. Green line represents the HDMI model, red line depicts HDMI-C model, and the blue line corresponds to the C model. The table on the right side of the figure contains a summary of the test-set performance of the biomarkers in discriminating metastatic and reactive ALNs. HDMI = high-definition microvasculature imaging, HDMI-C = high-definition microvasculature imaging + clinical data, AUC = area under the curve, ROC = receiver operating characteristic.

The ROC curves for the HDMI, HDMI-C, and C models are shown in Fig. 5. All 15 HDMI biomarkers were included in the HDMI model. The AUC for this model in the leave-out test set (containing 10 samples from the reactive set and 10 samples from the metastatic set) was 0.89 (95% CI [0.81,0.97]) with a sensitivity, specificity, and accuracy of 90%, 75%, and 83%, respectively. The AUC was further increased for the HDMI-C model. The corresponding AUC estimate was 0.90 (95% CI [0.82,0.98]) with the sensitivity, specificity, and accuracy of 90%, 88%, and 89%, respectively. The C model trained only on the clinical biomarkers had an AUC of 0.86 (95% CI [0.77,0.95]) with the sensitivity, specificity, and accuracy of 80%, 80%, and 80%, respectively.

## Discussion

The current study explored the performance of the quantitative biomarkers of contrast-free high-definition microvessel imaging (HDMI) in differentiating metastatic and reactive ALNs**.** Our findings reveal that most of the HDMI biomarkers (Tortuosity, Diameter, NV, NBP, VD, mvFD, MD, BA and VDR) demonstrated significant differences between metastatic and reactive ALNs. In the current study, the SVM-based classification models demonstrated the discriminating performance of using HDMI biomarkers as training features. SVM is known to be effective for classification of high-dimensional data, and in conditions with limited number of samples compared to the number of features. The addition of the clinical features age, cortical thickness, lymph nodes size and BI-RADS scores, to the multivariable analysis can increase the sensitivity, accuracy, and AUC (as was the case for our test set in this study).

Preclinical studies using contrast enhanced ultrasound approach [44] and 3-dimensional super resolution imaging of microvessels of rabbit’s lymph node [45] have examined the importance of microvessel density for diagnosis of lymph node metastasis, however, both techniques need the injection of exogenous contrast agents, and have limited quantification evaluation. Without the help of contrast agents, a clinical study was conducted using superb microvessel imaging (SMI) [27] for differentiation of metastatic from reactive lymph nodes, however, the quantification was limited to vessel index estimation and pixel counting with a sensitivity and specificity of and 69% and 63%, respectively. Instead, the current work combines a wide range of quantitative HDMI biomarkers that can separate metastatic from reactive ALN with higher sensitivity and specificity than what was reported in the SMI study. The further advantage of the proposed method is that the enhancement and visualization of microvessels at the submillimeter level is possible without the help of contrast agents. Additionally, HDMI method can accurately quantify vessel diameter, which may be challenging in contrast-enhanced tracking approaches [46].

The current study shows increased numbers in vessel segments and branch points in metastatic lymph nodes, indicating the presence of a greater level of vessel sprouting, an important marker in metastatic lymph nodes [47]. This result is supported by previous studies [30, 31, 48]. Vessel tortuosity metric was also statistically significantly higher in metastatic compared to reactive ALNs, which is in agreement with the fact that vessel tortuosity analysis can offer additional information that help with discriminating malignant lesions from hypervascular benign lesions [49]. Our study also shows maximum vessel tortuosity has higher statistically significant differences compared to averaging. This is in agreement with prior studies that have shown that as tumor expands in size, more tortuous vessels with increased diameter are seen in the periphery rather than the center of the tumor [22, 50], suggesting that averaging these biomarkers has less diagnostic value than their maxima. The current study also investigated the performance of other vessel morphological parameters such as Murray’s deviation, showing a significantly higher value in metastatic ALNs. This is supported by observation of deviation from Murray’s Law, observed in diseased tissue and for differentiation of malignant from benign breast masses, reported in [30, 31, 48]. Furthermore, current study shows statistically significant differences of bifurcation angle in metastatic and reactive ALNs that is also observed in breast masses differentiation [30, 31, 48]. In current study, microvascular complexity, measured by mvFD, shows higher values in metastatic compared to reactive ALNs. This finding indicates that malignant lesions tend to have dense vascularity, with irregularly branched and twisted microvessels, therefore the findings indicate promise, but that would need to be followed with additional studies[30, 31, 51].

One limitation of our study is that the imaging method used in this study is two-dimensional (2D). Estimating HDMI biomarkers in 2D plane may ignore some of the morphological features and the connectivity of vessels in three-dimensional (3D) space, resulting in either underestimation or overestimation of many morphometric parameters [52]. Additionally, the sample size was relatively small. To address these limitations, we plan to advance our 3D-HDMI technique and study on a large patient population to further investigate the efficiency of these morphometric parameters in differentiating metastatic ALNs from reactive.

## Conclusion

The use of a noninvasive and affordable quantitative imaging tool that provides objective information is of importance for prediction of metastatic ALNs in breast cancer patients. In current study, we showed the performance of quantitative vessel biomarkers extracted by a contrast-free ultrasound microvessel imaging technique, HDMI. Using the morphological features of tumor microvessels, we were able to separate metastatic from reactive ALNs with high accuracy. Addition of clinical factors to the set of features showed a potential for further increase of the classification accuracy in this study. Furthermore, as a complementary tool to conventional ultrasound, quantitative HDMI could offer a way to accurately assess the status of ALNs, staging breast cancer, and minimize the number of unnecessary ALN biopsies.

## Data Availability

The data that support the findings of this study are available from the corresponding author upon reasonable request. The requested data may include figures that have associated raw data. Because the study was conducted on human volunteers, the release of patient data may be restricted by Mayo policy and needs special request. The request can be sent to: Karen A. Hartman, MSN, CHRC | Administrator—Research Compliance| Integrity and Compliance Office | Assistant Professor of Health Care Administration, Mayo Clinic College of Medicine & Science | 507–538-5238 | Administrative Assistant: 507-266-6286 | hartman.karen@mayo.edu Mayo Clinic | 200 First Street SW | Rochester, MN 55905 | mayoclinic.org. We do not have publicly available Accession codes, unique identifiers, or web links.

## Code availability

The custom code or mathematical algorithms that are deemed central to the conclusions are available from the corresponding author upon request.
